# Plasma Cotinine Is Positively Associated with Homocysteine in Smokers but Not in Users of Smokeless Tobacco

**DOI:** 10.3390/ijerph182111365

**Published:** 2021-10-29

**Authors:** Elisabet Söderström, Torbjörn K. Nilsson, Jörn Schneede, Per-Magne Ueland, Øivind Midttun, Björn Gylling, Ingegerd Johansson, Johan Hultdin

**Affiliations:** 1 Department of Medical Biosciences, Clinical Chemistry, Norrbotten County Council, Sunderby Hospital, Umeå University, SE 90187 Umeå, Sweden; 2 Department of Medical Biosciences, Clinical Chemistry, Umeå University, SE 90187 Umeå, Sweden; torbjorn.nilsson@umu.se (T.K.N.); johan.hultdin@umu.se (J.H.); 3 Department of Pharmacology and Clinical Neuroscience, Clinical Pharmacology, Umeå University, SE 90187 Umeå, Sweden; jorn.schneede@umu.se; 4 Department of Clinical Science, University of Bergen and Laboratory of Clinical Biochemistry, Haukeland University Hospital, N 5021 Bergen, Norway; per.ueland@ikb.uib.no; 5 Bevital AS, N 5021 Bergen, Norway; Bjorn.Midttun@uib.no; 6 Department of Medical Biosciences, Pathology, Umeå University, SE 90187 Umeå, Sweden; bjorn.gylling@umu.se; 7 Department of Odontology/Section of Cariology, Umeå University, SE 90187 Umeå, Sweden; ingegerd.johansson@umu.se

**Keywords:** cotinine, nicotine, homocysteine, tobacco

## Abstract

Plasma total homocysteine (tHcy) is a risk marker, and smoking is an established risk factor for cardiovascular disease. It is unclear if the effect of smoked tobacco on homocysteine is mediated by nicotine or other combustion products in smoked tobacco. Snus (moist smokeless tobacco) is high nicotine-containing tobacco, and little is known about the effect of snus on plasma homocysteine. Therefore, we studied, in a cross-section of subjects (n = 1375) from the Northern Sweden Health and Disease Study, with strictly defined current smokers (n = 194) and snus users (n = 47), the impact of tobacco exposure on tHcy, assessed by self-reported tobacco habits and plasma cotinine concentrations. The snus users had higher cotinine concentrations than the smokers. Cotinine, creatinine, methylmalonic acid, and the methylenetetrahydrofolate reductase genotype (*MTHFR*) T allele were positively associated with tHcy among the smokers, but not among the snus users. No association was observed between tHcy and the number of cigarettes/day. There was a positive association between cotinine and tHcy in the smokers, but not among the snus users. This indicates that substances other than nicotine in tobacco smoke could be responsible for the differential effects on homocysteine status. Self-reported smoking should be complemented by a cotinine assay whenever possible.

## 1. Introduction

Plasma total homocysteine (tHcy) is primarily considered to be a biomarker of functional vitamin B12 and folate status, and has also been demonstrated to be useful as a general risk marker of cardiovascular disease (CVD) [1,2]. tHcy is associated with cardiovascular risk factors [3] and correlates with the Framingham cardiovascular risk score [4]. Smoking is one of the strongest established risk factors for CVD. However, studies have shown no increased risk of ischemic heart disease (IHD) for smokeless tobacco, but an increased risk of IHD deaths [5]. Furthermore, assessing smoking status by self-reported consumption is problematic due to reporting bias, and measuring the nicotine metabolite cotinine in plasma may serve as an objective method of ascertaining nicotine exposure [6]. Cotinine is stable during long-term storage [7], making it suitable as a marker of smoking status in biobank material. Cotinine has been demonstrated to be elevated not only in smokers, but also in snus users. Snus is a Swedish term for moist smokeless tobacco. The nicotine content/g tobacco is lower in snus compared to cigarettes, but the bioavailability is higher, and the observed plasma cotinine concentrations are often reported to be similar or even higher in snus users than in smokers [8,9,10]. Nicotine has direct hemodynamic effects, which may contribute to cardiovascular risk, but tobacco smoke contains more than 7000 chemical compounds in addition to nicotine. Smoking, therefore, has multiple and diverse potentially toxic effects, and may also influence tHcy through different mechanisms. Smokers have been reported to have higher tHcy [4,11,12,13,14] and lower concentrations of some B vitamins, including vitamin B12 [15] and folate [14]. The lower levels of folate in smokers may reflect a lower dietary intake of vegetables than non-smokers [16]. Little is known about the effects of smokeless tobacco on tHcy and if the effect of smoking on tHcy is mediated by nicotine or other compounds.

This study’s primary aim was to investigate if smoking is a stronger determinant for tHcy in plasma than the smokeless tobacco product snus after adjustment for potential confounders. We also wanted to explore whether plasma cotinine concentrations were a better predictor of tHcy concentrations than self-reported smoking data.

## 2. Materials and Methods

### 2.1. Study Population

The population originates from the Västerbotten Intervention Project (VIP) within the Northern Sweden Health and Disease Study (NSHDS) [17]. In VIP, initiated in 1985 and still ongoing, the majority of residents of Västerbotten are invited to a health survey at their primary health care center the year they turn 30, 40, 50, and 60 years old (since 1996 at 40, 50, and 60 years of age). All participants take part in a medical examination, complete a standardized questionnaire, including lifestyle factors, and donate a fasting blood sample to the Northern Sweden Medical Biobank for future research. As of 31 December 2009, the current study’s cut-off date, the VIP had collected 115,147 blood samples from 85,877 individuals. There is no mandatory folate fortification in this population, and those with diseases influencing tHcy, especially reduced renal function, are rare in this cohort. Selection bias has been reported to be minimal [18]. In this study, we used a cross-sectional design in a large number of subjects in whom an extensive set of metabolites and biomarkers, including cotinine, had been analyzed. Subjects were initially selected for prospective nested case-referent studies on colorectal cancer, case-referent ratio 1/2 [19,20]. The cases developed colorectal cancer years later (median follow up time 8.2 years) after inclusion in the study and donating blood. Study subjects with a previous cancer diagnosis, other than non-melanoma skin cancer, insufficient stored plasma sample volumes, or prioritized to other studies were excluded. Thus, our sample consists of 1375 subjects.

In this population, data on self-reported snus use and cigarette smoking were available. One hundred and seventy subjects could not be categorized because of missing data on tobacco use. Subjects that were both snus users and smokers (n = 20) were excluded from the groups of snus users and smokers, as were former snus users, former smokers, and occasional smokers (n = 392), thus we had strictly defined 194 current smokers and 47 current snus users. Never tobacco users included subjects who had never smoked or used snus (n = 479) and those who formerly had only been occasional smokers (n = 73). Subjects with cotinine plasma concentrations above the cut-off for passive smokers (>85 nmol/L, n = 12) were excluded from the group of never tobacco users, resulting in a group of 540 strictly defined never tobacco users.

The study was conducted according to the guidelines of the Declaration of Helsinki, and approved by The Research Ethics Committee of Umeå University, Umeå, and the National Computer Data Inspection Board approved the data handling procedures (dnr 03-186).

### 2.2. Blood Sampling and Laboratory Procedures

In VIP, peripheral venous blood specimens were collected in evacuated EDTA-containing test tubes. Sixty percent of the study subjects had fasted for more than 8 h, 17% had fasted 4–8 h, and 23% had fasted less than 4 h. After centrifugation at 1500× *g* for 15 min, plasma was aliquoted and stored at −80 °C until analysis. Bevital AS (Bergen, Norway) performed the biochemical analyses. Total plasma homocysteine (tHcy) was determined by an isotope dilution gas chromatography–mass spectrometry method (GC–MS) [21]. Cotinine and creatinine concentrations were measured with liquid chromatography/tandem mass spectrometry (LC-MS/MS) [22,23]. The limit of detection for cotinine was 1.0 nmol/L (0.18 ng/mL). Plasma folate and vitamin B12 were analyzed with a microbiological assay, using *Lactobacillus casei* to determine plasma folate [24] and *Lactobacillus leichmannii* for measuring plasma vitamin B12 [25]. The single nucleotide polymorphism *MTHFR* 677C>T (rs1801133) was determined by MALDI-TOF mass spectrometry [26].

The within-day coefficient of variation (CV) and between-day CV were 0.9% and 2.1% for tHcy, 2–3% and 6% for cotinine, 4% for creatinine, and 4% and 5% for both folate and vitamin B12, respectively. The average error rate of genotyping for the *MTHFR* 677C>T polymorphism was ≤0.1%. The samples were analyzed in triplets consisting of two referents and one case. To avoid systemic bias, laboratory staff were blinded to all information about the study subjects.

### 2.3. Statistical Analysis

Calculations were performed using IBM SPSS Statistics 26 (IBM Corporation, New York, NY, USA). Hardy–Weinberg equilibrium for the *MTHFR* 677C>T genotypes was calculated based on the χ^2^ test. In all subjects, cotinine categories were defined as <5 = I (n = 831), 5–85 = II (n = 94), >85–1700 = III (n = 332), and >1700 nmol/L = IV (n = 118), according to Bevital´s in laboratory cut-offs. When analyzing the subgroups of smokers and snus users, cotinine had a Gaussian distribution within these two sub-groups and was included in the model as a continuous variable (Additional file 1). Consumption of cigarettes was categorized into the following four groups according to number of cigarettes daily: 1–4 (n = 25), 5–14 (n = 88), 15–24 (n = 47) and ≥25 (n = 9). BMIs were categorized according to WHO, <25, 25–30 and >30 kg/m^2^.

To compare variables between never tobacco users, smokers, and snus users, we used Mann–Whitney two independent samples test for continuous, and χ^2^ test for categorical variables. To explore associations between the biochemical variables and lifestyle characteristics, we calculated Spearman’s rho. Due to skewed distributions, tHcy, folate, and methylmalonic acid (MMA) were logarithmically transformed and included in the linear univariate regression model. We selected tHcy (log) as the dependent variable in the multiple regression models, where we included potential confounders and significant variables from the univariate analyses. MMA in plasma is mainly determined by bioavailable vitamin B12 and we therefore omitted vitamin B12 in the multiple analyses. The adjusted coefficient of determination *R^2^* was used to estimate the degree of linear correlation in the complete models. Partial Eta squared was used to determine the effect size for the corrected models and the individual variables included in the models. We performed a sensitivity analysis for case-control status (cases later developed colorectal cancer) [19].

## 3. Results

The characteristics for all the subjects and subgroups, according to self-reported tobacco exposure, are presented in Table 1. The cotinine concentrations were less than 74 nmol/L among the never tobacco users, and both the smokers and snus users had high concentrations, with the highest concentrations being observed in the latter group. Distributions of plasma cotinine concentrations are also shown in Supplementary Material Figures S1–S3. Other notable observations were lower folate concentrations and a trend towards higher tHcy in the smokers compared to the non-smokers. The median values for plasma concentrations of tHcy and folate in the snus users did not differ from the non-smokers. Further, 98.9% and 95.5% of the subjects had creatinine and MMA concentrations below 100 µmol/L and 0.26 nmol/L, respectively.

Cotinine was positively associated with tHcy when all the subjects were combined and investigated, though the strongest association was observed among the smokers. In the snus users, on the other hand, the association between cotinine and tHcy was found to be close to zero (Table 2). No association was evident between tHcy and the number of cigarettes/day for all types of smokers among all the subjects or in the defined groups of smokers. Other findings in the same groups were negative associations between tHcy and plasma vitamin B12 and folate concentrations, respectively, while positive associations were observed for tHcy with MMA and creatinine. The *MTHFR* 677 T allele showed a positive association in all the subjects with tHcy in codominant, recessive, and dominant models; since the recessive model showed the strongest association, the data are only included for this model (Table 2). In the snus user group, we observed no statistically significant correlations between any biomarkers or *MTHFR* 677 T alleles with tHcy.

In the multiple regression analyses using the main effects model (Table 3), cotinine remained positively associated with tHcy (log) in all the subjects and among the smokers. In contrast, no association was observed among the snus users. In the same model, among the snus users, the only significant finding was a negative association between tHcy and folate (log). Between homocysteine and the *MTHFR* 677 polymorphism, an association was found in the smokers, but not in the snus users. In a sensitivity analysis, adding a case-control status to the multiple regression models, the results did not change. The case-control status was not significantly associated with tHcy (data not shown).

## 4. Discussion

In the present study, including 1375 subjects, users of the smokeless tobacco snus had higher cotinine concentrations than the smokers. Despite this, the never tobacco users, smokers, and snus users did not have different tHcy concentrations. However, we found a positive association between cotinine and tHcy among the smokers. Among the snus users, the association was close to zero in the model. For the smokers, this is in line with the results from the NHANES study, where cotinine was an independent marker for tHcy > 14 µmol/L. [27]. We are not aware of any previous studies on the association between cotinine and tHcy in users of the smokeless tobacco snus.

In line with our study, higher plasma cotinine concentrations have been previously reported in snus users compared to smokers [9] and other smoking forms, including e-cigarettes [10]. Heated tobacco products, other than e-cigarettes, have also been introduced into the market. Differences in the clinical risk markers for cardiovascular and respiratory diseases have been noted in a recent review, where users of heated tobacco products had a better biomarker profile compared to persistent smokers [28]. One study reported no short-term changes in tHcy in smokers using conventional cigarettes, randomized to smoking abstinence, menthol cigarettes, or menthol tobacco heating systems (n = 40, 42, and 78, respectively) [29]. We had no subjects using heated tobacco products in our study. The finding of similar tHcy concentrations in the different tobacco exposure groups is in contrast with previous studies comparing smokers and non-smokers [11,12,15].

In our study, as in many studies based on biobank samples, smoking status is based on self-reported data from questionnaires completed at the time of blood sampling. The self-reported number of cigarettes did not correlate with tHcy. In comparison, the plasma cotinine concentrations showed a positive correlation with tHcy, both in smokers and in all the subjects combined (including all combinations of previous, current smoking, and or snus use), which points to the advantage of using objective methods for ascertaining nicotine exposure. Reporting bias is well known from previous studies [30], and our data underscore the importance of assessing smoking status by biomarker assays to prevent erroneous conclusions about pathophysiological processes.

We found that the median folate concentrations were higher in never tobacco users than among smokers and snus users, with the two groups of tobacco users having similar median folate concentrations. However, only the difference between smokers and never tobacco users reached statistical significance, presumably due to the smaller snus user group. The lower folate concentrations among the smokers are consistent with previous studies [13,14,31,32,33]. Regarding smokeless tobacco users, so far, only the NHANES 1999–2008 study has reported on folate in a limited number of subjects (n = 69), defined as a mixed group of chewing tobacco and moist snuff users [13]. In line with our findings, they reported lower folate concentrations among smokeless tobacco users than non-tobacco consumers, with no difference in folate between smokeless tobacco users and smokers. Although, for snus users vs. non-tobacco users, the difference did not reach statistical significance in our study.

Between the groups, the vitamin B12 concentrations did not differ, which is in line with a previous study [14]. Similarly to earlier studies, we found comparable MMA concentrations among smokers and never tobacco users [14,15,31]. The creatinine concentrations were lower among smokers than never tobacco users. One reason for this could be the higher age in the latter group (median: 50.3 vs. 59.7 years). Another reason could be the hyperfiltration of creatinine caused by smoking [34].

We found a moderately strong positive correlation between cotinine and tHcy (r = 0.27) among the smokers. This correlation is consistent with a previous study analyzing cotinine in urine [15], and with the NHANES study analyzing cotinine in serum [27]; although, the latter study had no data on the *MTHFR* genotype. On the other hand, among the snus users, a null association was found between cotinine and tHcy. Multiple regression analyses with adjustments for possible confounders confirmed this. Among the snus users, the only significant univariate association of tHcy was with folate, also after multiple adjustments. The fact that MMA was higher among the never tobacco users compared to the smokers is somewhat contra-intuitive. Our data do not support the hypothesis of an impaired B12 status in smokers, but minor differences could represent the age difference between the groups. The never tobacco users were older and had higher creatinine values, indicating slightly lower renal function, resulting in marginally higher MMA values. The different findings among the snus users and smokers may indicate that cotinine in itself is not causally associated with tHcy. Instead, substances other than nicotine, probably combustion products in tobacco smoke, mediate the effect on the homocysteine status among smokers. An example of such a combustion product is urinary thiocyanate, a biomarker of smoked tobacco formed from cyanide. Thiocyanate is elevated in smokers versus non-smokers, but is lower in smokeless tobacco users than smokers [35]. It has been shown that cyanide induces the formation of cyanocobalamin, which lacks enzymatic function. Furthermore, hydrogen sulfide exposure results in inactive sulfocobalamin [31]. Thus, the higher tHcy concentrations in smokers than non-smokers may also partly be attributed to inadequate functional vitamin B12 status [15]. The lack of association between cotinine and tHcy among the snus users may also apply to other forms of smokeless tobacco. Users of e-cigarettes have similar cotinine concentrations in plasma as smokers [10,36]. Still, the results on smokeless tobacco in this study cannot be transferred to users of e-cigarettes without further studies, as there are many different substances inhaled from e-cigarettes, and the inhaled products are combusted [37].

## 5. Strengths and Limitations

This study’s main strength was the population-based study design, which was homogenous regarding ethnicity and representative of the population of Northern Sweden. We evaluated the effects of tobacco on tHcy by using both self-reported tobacco habits and plasma cotinine concentrations as a more objective measure of nicotine exposure. All the samples were analyzed in one batch, reducing time-dependent methodological variability. We were also able to adjust for other determinants for tHcy, including the *MTHFR* 677C>T polymorphism. This study’s main limitation was that, since the dual use of both snus and smoking is very common, we had a relatively low number of subjects exclusively using snus. The other limitations are that no other compounds in cigarette smoke, such as thiocyanate, were measured. The fact that the population was originally a case-referent cohort could be a possible limitation, but the median follow-up time from blood sampling was 8.2 years, reducing the risk for reversal causation, and homocysteine has not previously been observed to be associated with colorectal cancer risk in this cohort [19,38]. Also, including a case-control status in a sensitivity analysis did not change the results.

## 6. Conclusions

We found a positive association between cotinine concentrations and tHcy among smokers, but not among snus users. The differential associations between cotinine and tHcy in smokers and snus users indicate that nicotine per se may not be a causal factor; instead, other substances formed from combusted tobacco may affect tHcy. Whenever possible, self-reported smoking should be complemented by biomarker assays, such as cotinine, in epidemiological studies.

## Figures and Tables

**Table 1:** Characteristics presented as medians (25–75%) for continuous variables and as proportions/percent for categorical variables for all subjects with cotinine data, and strictly defined groups of never tobacco users, smokers and snus users.

Variables	All	Never Tobacco Users	Smokers	Snus Users	Smokers vs. Never Tobacco Users *p* ^a^	Snus Users vs. Never Tobacco Users *p* ^a^	Smokers vs. Snus Users *p* ^a^
n (male/female)	1375 (715/660)	540 (232/308)	194 (84/110)	47 (45/2)			
Age, years	59.5 (50.0–60.0)	59.7 (50.0–60.0)	50.3 (49.9–60.0)	50.4 (40.2–59.9)	**0.010**	**0.010**	NS
BMI, kg/m^2^	25.7 (23.5–28.1)	25.5 (23.3–28.0)	25.3 (22.5–27.8)	25.6 (23.9–27.8)	NS	NS	NS
SBP, mmHg	130 (120–145)	130 (120–145)	128 (115–140)	130 (118–140)	**0.006**	NS	NS
Cotinine, nmol/L	1.56 (<1.0–786)	<1.0 (<1.0–1.47)	1160 (772–1504)	1689 (1222–2459)	**<0.001**	**<0.001**	**<0.001**
Homocysteine, µmol/L	9.84 (8.25–11.53)	9.68 (8.10–11.17)	10.18 (8.12–12.17)	9.88 (8.33–11.07)	NS (0.057)	NS	NS
Vitamin B12, pmol/L	416 (342–500)	417 (340–503)	420 (335–482)	389 (346–473)	NS	NS	NS
Folate, nmol/L	7.20 (4.77–10.31)	7.76 (4.98–10.65)	6.65 (4.71–9.18)	6.59 (4.28–9.83)	**0.016**	NS	NS
Creatinine, µmol/L	66.3 (58.0–74.4)	67.0 (58.2–75.0)	62.3 (53.7–68.8)	74.7 (69.8–82.2)	**<0.001**	**<0.001**	**<0.001**
MMA, µmol/L	0.14 (0.12–0.17)	0.15 (0.12–0.17)	0.14 (0.12–0.16)	0.14 (0.12–0.19)	**0.043**	NS	NS
*MTHFR* 677C>T ^b^							
CC	49.9	50.4	51.0	50.0	NS	NS	NS
CT	40.6	41.3	38.0	43.5	NS	NS	NS
TT	9.4	8.3	10.9	6.5	NS	NS	NS

^a^ *p*-values calculated with Mann–Whitney two independent samples test for continuous variables and by chi-square test for independence for categorical variables. ^b^ The *MTHFR* 677 genotypes were in Hardy–Weinberg equilibrium. Significant *p*-values are shown in bold. BMI = body mass index, SBP = systolic blood pressure, NS = not significant.

**Table 2:** Spearman’s rho correlations between tHcy and other variables.

Variables	All	N	Smokers	N	Snus Users	N
Sex	−0.220 ^a^	(1375)	−0.228 ^a^	(194)	−0.171	(47)
Age	0.173 ^a^	(1375)	0.149 ^b^	(194)	0.228	(47)
BMI ^c^	0.116 ^a^	(1354)	0.024	(188)	−0.025	(47)
Number of cig/day ^d^	0.084	(311)	0.149	(169)	n.a.	(47)
Cotinine ^e^	0.098 ^a^	(1375)	0.270 ^a^	(194)	−0.008	(47)
Vitamin B12	−0.255 ^a^	(1359)	−0.348 ^a^	(194)	0.001	(47)
Folate	−0.366 ^a^	(1375)	−0.277 ^a^	(194)	−0.276	(47)
Creatinine	0.268 ^a^	(1372)	0.280 ^a^	(193)	0.168	(47)
MMA	0.213 ^a^	(1375)	0.245 ^a^	(194)	−0.017	(47)
*MTHFR* 677 rec ^f^	0.170 ^a^	(1358)	0.197 ^a^	(192)	0.063	(46)

^a^*p* < 0.01. ^b^ *p* < 0.05. ^c^ BMI categorized, as follows, according to WHO: <25, 25–<30, ≥30 kg/m^2^. ^d^ Number of cigarettes: 1–4, 5–14, 15–24, ≥25 cigarettes/day. ^e^ In all subjects (including all combinations of former, current smoking, and or snus use) cotinine was categorized as follows: <5, 5–85, >85–1700, >1700 nmol/L; in smokers and snus users cotinine was used as a continuous variable. ^f^ *MTHFR* 677 rec: 0 = 677 CC and CT, 1 = 677 TT (recessive model).

**Table 3:** Main effects multiple regression models on possible determinants of tHcy (log).

	All		Smokers		Snus Users	
Adjusted R^2^ Complete Model	0.345		0.292		0.115	
Variables	Partial Eta^2^	*p* ^a^	Partial Eta^2^	*p* ^a^	Partial Eta^2^	*p* ^a^
Corrected model	0.35	**1.5 × 10** ^**−115**^	0.326	**1.3 × 10** ^**−11**^	0.292	0.138
Sex	0.004	**0.028**	0.002	0.522	0.002	0.816
Age	0.013	**3.1 × 10** ^**−5**^	0.003	0.469	0.093	0.063
BMI ^b^	0.010	**0.002**	0.006	0.602	0.003	0.949
Cotinine ^c^	0.019	**1.0 × 10** ^**−5**^	0.029	**0.023**	0.006	0.655
Folate (log)	0.124	**4.5 × 10** ^**−40**^	0.077	**1.9 × 10** ^**−4**^	0.122	**0.031**
Creatinine	0.061	**9.9 × 10** ^**−20**^	0.035	**0.012**	0.001	0.838
MMA (log)	0.079	**1.6 × 10** ^**−25**^	0.085	**8.6 × 10** ^**−5**^	0.032	0.286
*MTHFR* 677 rec ^d^	0.052	**3.6 × 10** ^**−17**^	0.059	**0.001**	0.047	0.193

^a^ Significant *p*-values are shown in bold. ^b^ BMI categorized, as follows, according to WHO: <25, 25-<30, ≥30 kg/m^2^. ^c^ In all subjects (including all combinations of previous, current smoking, and or snus use) cotinine was categorized as follows: <5, 5–85, >85–1700, >1700 nmol/L; in smokers and snus users cotinine was used as a continuous variable. ^d^ *MTHFR 677* rec: 0 = 677 CC and CT, 1 = 677 TT (recessive model).

## Data Availability

Aggregate data are included in the manuscript and its Supporting Information files. Individual data are not publicly available, as they contain potentially identifying patient information. Data are available upon request from the Northern Sweden Health and Disease Study Biobank. To request data, interested researchers must complete a formal application (available at https://www.umu.se/en/biobank-research-unit/research/access-to-samples-and-data/access-to-nsdd/ (accessed on 28 October 2021) and submit it to The Biobank Research Unit at Umeå University (contact via ebf@umu.se).

## Supplementary Materials

The following are available online at https://www.mdpi.com/article/10.3390/ijerph182111365/s1: Figure S1: distribution of plasma cotinine concentrations among all subjects; Figure S2: distribution of plasma cotinine concentrations among self-reported smokers; Figure S3: distribution of plasma cotinine concentrations among self-reported snus-only users (i.e., non-smokers).
