# Supplementary material for: Plasma Cotinine Is Positively Associated with Homocysteine in Smokers but Not in Users of Smokeless Tobacco
Source: Int J Environ Res Public Health. 2021 Oct 29;18(21):11365. doi: 10.3390/ijerph182111365 (PMC8583682; doi:10.3390/ijerph182111365)
Supplement: Supplementary file 1 [file ijerph-18-11365-s001.zip › ijerph-1386634-supplementary.pdf]

International Journal of
Environmental Research and Public Health

MDPI

Supplementary Material

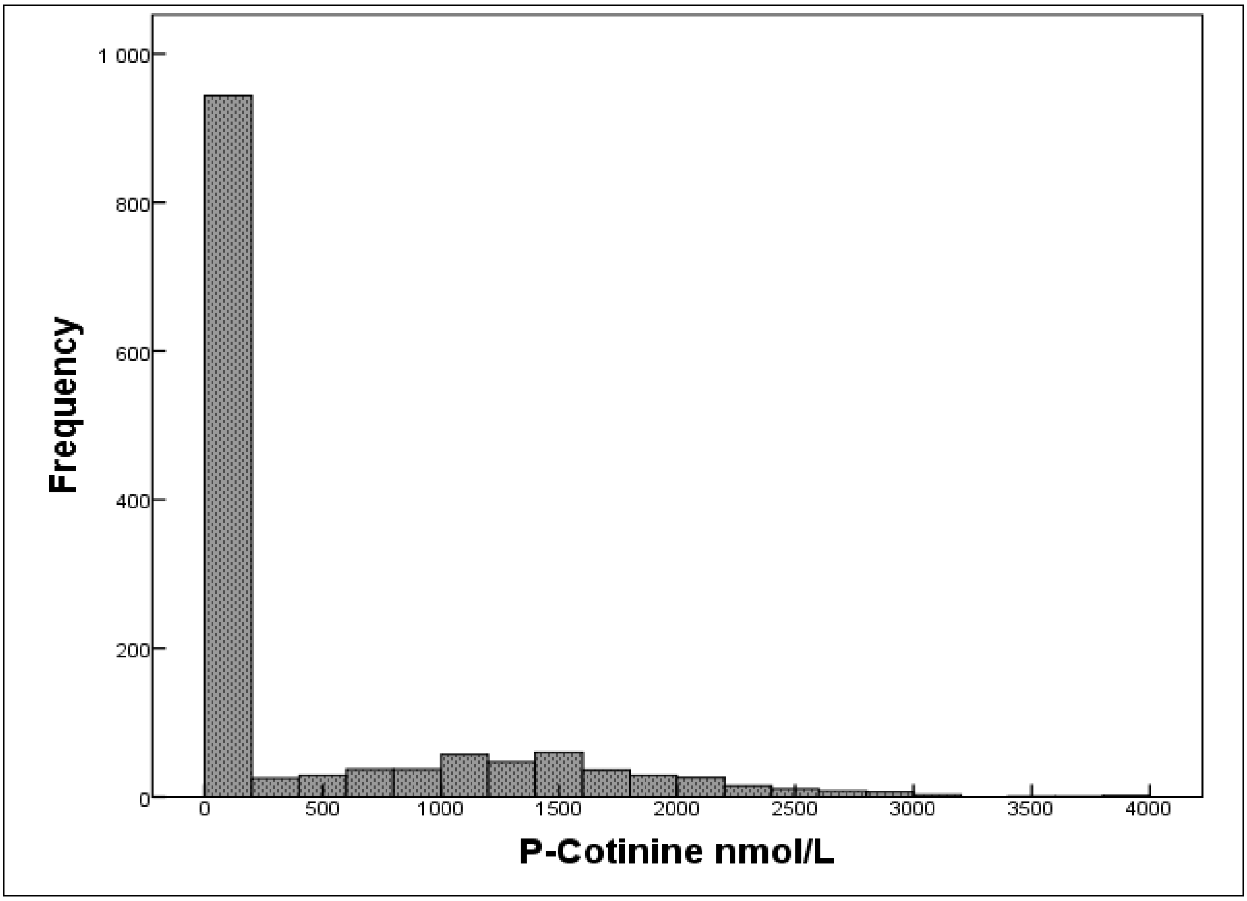

Figure S1. Distribution of plasma cotinine concentrations among all subjects.

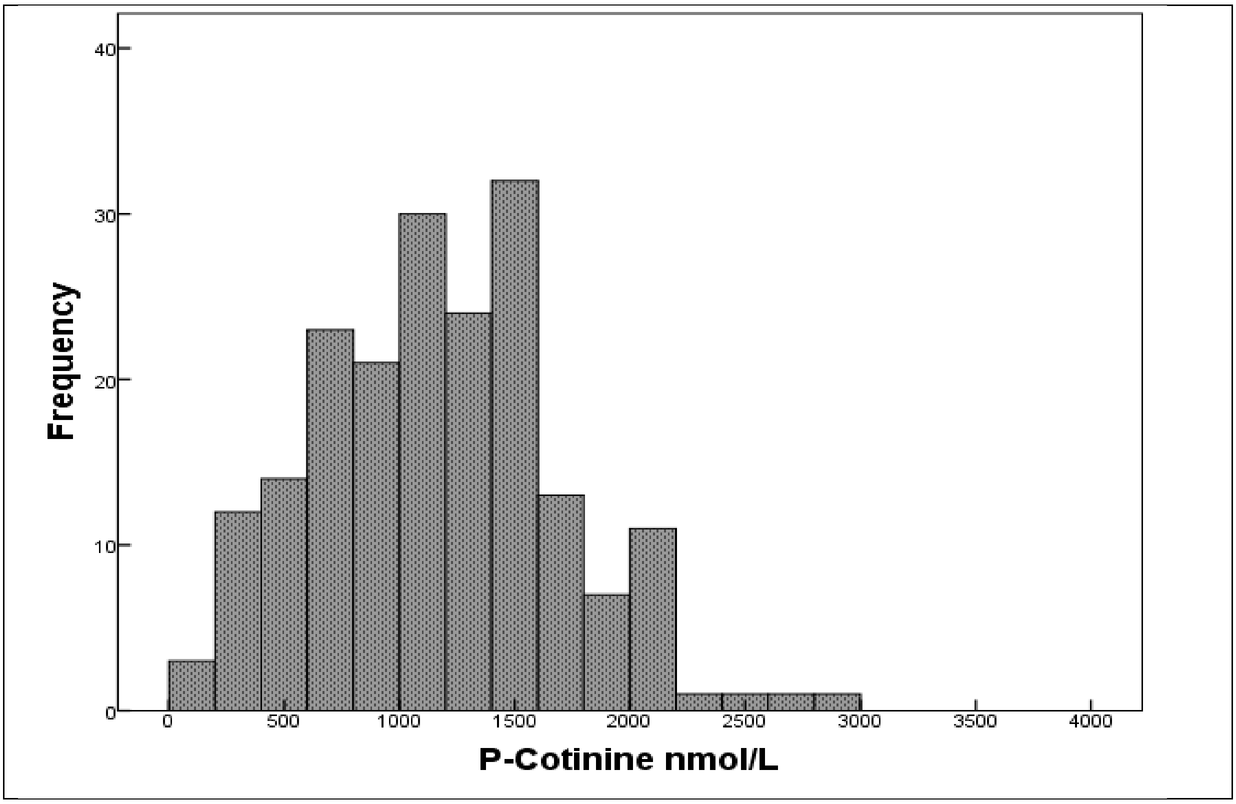

Figure S2. Distribution of plasma cotinine concentrations among self-reported smokers.

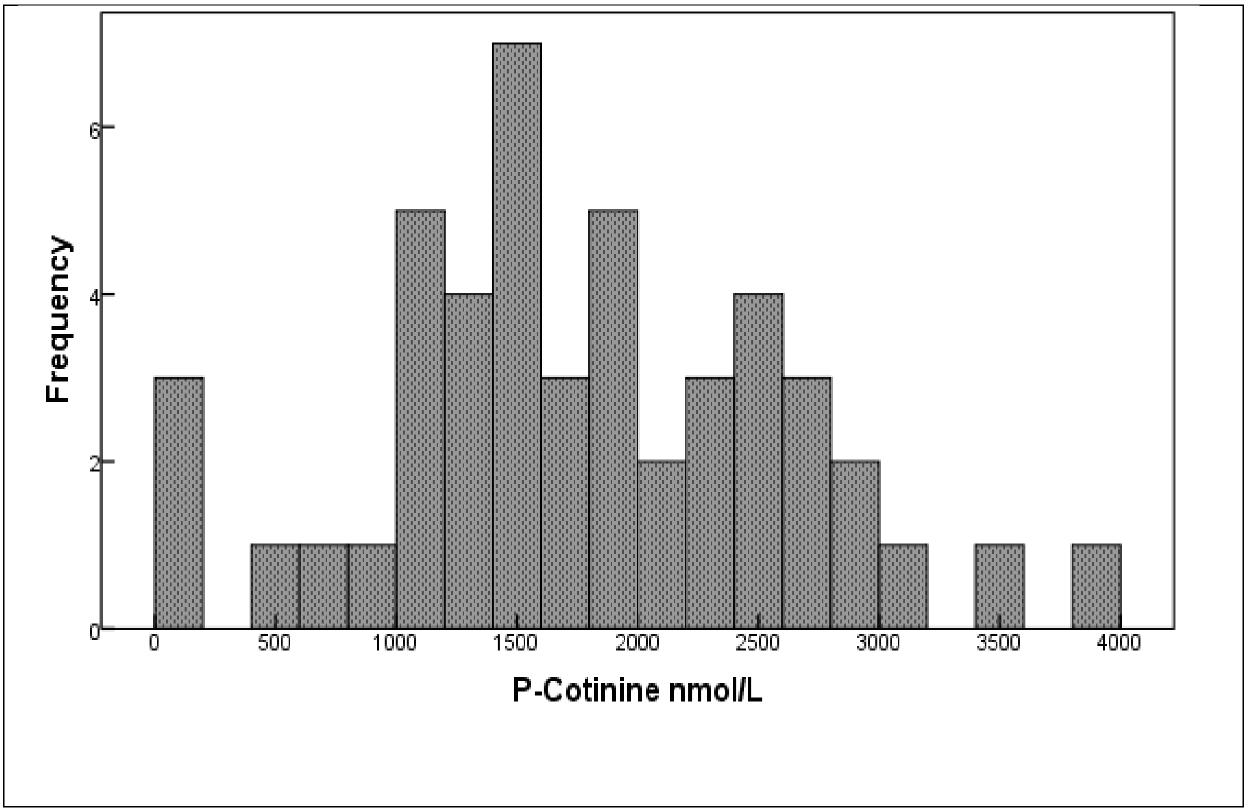

Figure S3. Distribution of plasma cotinine concentrations among self-reported snus-only users (i.e., non-smokers).
